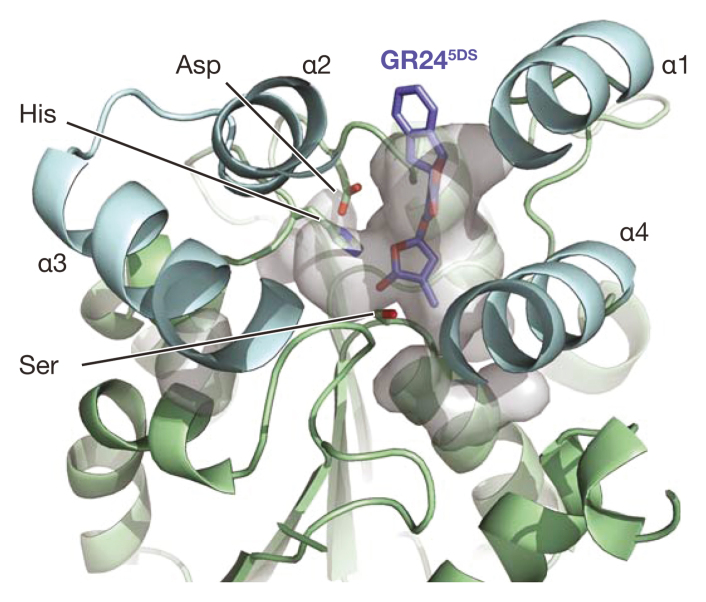# Perception of karrikins by plants: a continuing enigma

**DOI:** 10.1093/jxb/erz548

**Published:** 2019-12-14

**Authors:** Jiaren Yao, Mark T Waters

**Affiliations:** 1 School of Molecular Sciences, The University of Western Australia, Perth, WA, Australia; 2 University of Essex, UK

**Keywords:** Butenolide, development, hormone, karrikin, ligand, receptor, strigolactone

## Abstract

Karrikins are small butenolide molecules with the capacity to promote germination and enhance seedling establishment. Generated abiotically from partial combustion of vegetation, karrikins are comparatively rare in the environment, but studying their mode of action has been most informative in revealing a new regulatory pathway for plant development that uses the karrikin perception machinery. Recent studies suggest that the karrikin receptor protein KAI2 and downstream transcriptional co-repressors in the SMXL family influence seed germination, seedling photomorphogenesis, root morphology, and responses to abiotic stress such as drought. Based on taxonomic distribution, this pathway is ubiquitous and likely to be evolutionarily ancient, originating prior to land plants. However, we still do not have a good grasp on how karrikins actually activate the receptor protein, and we have yet to discover the assumed endogenous ligand for KAI2 that karrikins are thought to mimic. This review covers recent progress in this field, as well as current gaps in our knowledge.

## Teaser

The perception-response pathway for karrikins, small butenolide compounds found in smoke, influences seed germination, seedling photomorphogenesis, root morphology, and responses to abiotic stress such as drought.

## Introduction

Karrikins are a set of small butenolide compounds that were first reported as seed germination stimulants isolated from plant-derived smoke (Flematti *et al.*, 2004). Karrikins promote germination in a number of species, including Arabidopsis, which was initially surprising because Arabidopsis is not normally associated with fire-prone environments (Nelson *et al.*, 2009; Waters, 2017). Karrikins increase the sensitivity of both seeds and seedlings to light (Nelson *et al.*, 2010), and thus it has been proposed that karrikins serve as an adaptive chemical signal that improves seedling recruitment and establishment after fire events (Nelson *et al.*, 2012).

In recent years, it has become clear that plants use much the same molecular mechanism for perception and response to karrikins as they do for strigolactones (SLs), another class of butenolide signalling compound in plants (Morffy *et al.*, 2016; De Cuyper *et al.*, 2017; Waters *et al.*, 2017). SLs are carotenoid-derived phytohormones that regulate shoot and root architecture, and promote root symbiotic relationships, especially with arbuscular mycorrhizal fungi. In brief, both karrikin and SL perception pathways use a functionally distinct α/β hydrolase receptor protein, a common F-box protein, and one or more different members of a family of putative transcriptional co-repressor proteins. Superficially and taken individually, these striking commonalities between the perception and response pathways for two different types of compound—one an abiotic and exogenous chemical of limited occurrence, the other a ubiquitous endogenous hormone with diverse functions in plant development—do not make much sense. But when considered together, these common features are what have propelled karrikins from a curiosity of fire-prone ecosystems to the starting point for the discovery of unknown compounds with much wider functional significance.

In this review, we highlight some recent advances in our knowledge of how karrikin signalling operates and its functions in plant growth and development. We also discuss structural aspects of how karrikins interact with the karrikin receptor, and how ligand specificity between karrikin-like molecules and SLs might be achieved.

## Karrikins: what, and so what?

Karrkins are small (molecular mass ~150 Da), bicyclic compounds produced from the partial combustion of cellulose. Six karrikins (KAR_1_ to KAR_6_) have been isolated from samples of smokewater and subsequently confirmed by chemical synthesis, although most biological studies have used KAR_1_ and KAR_2_ (Flematti *et al*., 2004, 2007, 2009; Nelson *et al.*, 2009). Seed germination responses to karrikins vary widely among species, with some such as *Solanum orbiculatum* and *Lactuca sativa* responding to nanomolar levels of KAR_1_ (Flematti *et al.*, 2004, 2007). By contrast, some species—even those that are smoke responsive such as *Anigazanthos manglesii—*do not respond to karrikin at all, at least in terms of promoting germination (Flematti *et al.*, 2011). In the case of typical smoke-responsive species, KAR_1_ is the most bioactive karrikin (Flematti *et al.*, 2007), but this may reflect an adaptation to fire regimes rather than being a general truism for angiosperms, because Arabidopsis is more sensitive to KAR_2_ than KAR_1_ (Nelson *et al.*, 2009; Waters *et al.*, 2012). Unfortunately, relatively few studies have compared the response of non-smoke-responsive species to different KARs; doing so would help resolve the ancestral and derived states for karrikin preference.

The study of mutants has greatly enhanced our understanding of the function and bioactivity of particular compounds. In particular, the role of strigolactones as plant hormones was realized only through the study of shoot branching mutants (Gomez-Roldan *et al.*, 2008; Umehara *et al.*, 2008). In Arabidopsis, karrikin-insensitive mutants such as *kai2* or *max2* show sluggish germination (increased primary dormancy) and defective seedling photomorphogenesis (Nelson *et al.*, 2011; Sun and Ni, 2011; Waters *et al.*, 2012). Given that these phenotypes are the opposite of that resulting from applying karrikins, they are not all that surprising. However, it is the additional, less obvious phenotypes of karrikin-insensitive mutants—some of which are only observed in species other than Arabidopsis—that have broadened our appreciation of what karrikins can do. But crucially, it is the endogenous function of the KAI2 signalling pathway through which karrikins operate that tells us the most, rather than the function of karrikins specifically. A number of recent publications have expanded our knowledge of what KAI2-mediated signalling can do (see Box 1). There is now evidence that this pathway affects response and tolerance to abiotic stress, and is required for normal root development. An exciting breakthrough for KAI2-dependent signalling came in 2015 with the identification of a *kai2* mutant in rice that could not support arbuscular mycorrhizal symbiosis (Gutjahr *et al.*, 2015). It remains to be seen how widespread this role for KAI2 is among the very wide range of species that accommodate such symbioses. Likewise, one of the most promising roles for KAI2 is that of response to drought (Li *et al.*, 2017). To date, this role has only been reported using Arabidopsis mutants, but *kai2* knockouts in more species are becoming available (e.g. Carbonnel *et al.*, 2019, Preprint). Should the role in drought tolerance be conserved, this knowledge opens up exciting prospects for crop improvement strategies.

It is not realistic that a highly conserved signalling pathway for karrikins should exist when karrikins themselves are rare in the environment. Instead, karrikins are bioactive because they are able to hijack a pre-existing signalling mechanism with another purpose. This signalling pathway depends upon the karrikin receptor, KAI2, and downstream partner proteins SMAX1 and SMXL2 in Arabidopsis (see Box 2). Abundant phylogenetic evidence suggests that the canonical SL signalling pathway originated from the KAI2 signalling pathway (Bythell-Douglas *et al.*, 2017; Walker *et al.*, 2019). Combined with the pleiotropic developmental phenotypes of *kai2*, *max2*, and *smax1 smxl2* mutants, it seems clear that the normal function of KAI2 is to perceive an unknown endogenous butenolide ligand in a manner analogous to D14 and SLs. Indirect experimental evidence for KL (‘KAI2 ligand’) includes the fact that plant extracts can activate a KAI2-dependent transcriptional reporter (Sun *et al.*, 2016). Furthermore, KAI2 homologues from *Selaginella moellendorffii* and *Striga hermonthica* can complement the Arabidopsis *kai2* mutant phenotype without conferring responses to karrikins (Conn *et al.*, 2015; Waters *et al.*, 2015*b*), suggesting that these homologues retain the ability to perceive an endogenous ligand. Although the biosynthetic source of KL is a completely open question, it probably is not a SL derivative, because the SL precursor carlactone does not effectively trigger KAI2-dependent responses, and SL-deficient mutants of Arabidopsis do not exhibit *kai2*-like phenotypes (Shen *et al.*, 2012; Scaffidi *et al.*, 2013). Furthermore, two independent forward genetic screens for *kai2*-like phenotypes recovered 13 additional *kai2* alleles, but no KL biosynthesis mutants (Yao *et al.*, 2018). Therefore either there is some genetic redundancy in KL production, or KL is produced non-enzymically, or KL is an essential metabolite that signals through KAI2 but has other functions as well.

## How do similar receptor proteins distinguish similar ligands?

**Fig. 1.**
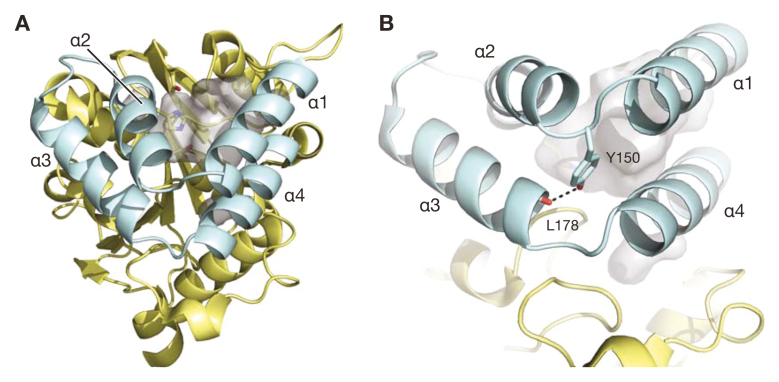
Structural details of Arabidopsis KAI2. (A) The overall structure of AtKAI2. The lid domain (cyan) comprises four α helices (α1 to α4) that sit atop the core domain (yellow). The side chains of the catalytic Ser, His, and Asp residues are visible at the bottom of the ligand binding pocket (grey surface). The pocket entrance is in the cleft between α1 and α2. (B) Close up view of the hydrogen bond (dashed line) between the side chain of Tyr150 and the main chain carbonyl group of Leu178. This bond is thought to stabilize the lid domain and could contribute to pocket size and ligand specificity (Xu *et al.*, 2018). Images created in PyMol v2.2.0 and based on PDB code 5Z9G (Lee *et al.*, 2018).

Considering the similarities between the KAI2- and D14-dependent signalling pathways, it is important to know how the two receptors perceive different ligands—not least because this information will allow precision targeting of one or both receptors by chemical means. Both proteins have a two-domain structure, which consists of a lid domain formed by two parallel V-shaped pairs of helices (α1/α2 and α3/α4), and a core domain consisting of seven α helices and seven β sheets (Fig. 1A). The two pairs of helices in the lid domain define a tunnel lined with hydrophobic residues that permits ligand access to the catalytic site in the core of the protein (Bythell-Douglas *et al.*, 2013; Guo *et al.*, 2013; Zhao *et al.*, 2013).

Natural variation among KAI2 homologues can help us understand how these proteins can distinguish karrikins and strigolactone ligands. Xu *et al.* (2018) examined 11 KAI2 proteins from *Striga hermonthica*, a root parasitic weed that uses KAI2 proteins to detect host-derived SLs (Conn *et al.*, 2015; Tsuchiya *et al.*, 2015). These KAI2 paralogues can be divided into three clades: a ‘conserved’ clade (ShKAI2-1) most similar to other angiosperm KAI2 proteins, an ‘intermediate’ clade (ShKAI2-2 and ShKAI2-3), and a ‘divergent’ clade (ShKAI2-4 to ShKAI2-11) with high affinity for SLs. Xu *et al.* (2018) found that the architecture of the pocket plays a vital role in ligand affinity. In general, KAR_1_-binding proteins have smaller pocket volumes than SL-binding proteins. The pairs of V-shaped helices restrict the tunnel size, and polar interactions stabilize these two sets of helices (Fig. 1B). The Y150 residue is located at the loop linking α1 and α2 of ShKAI2-1 and ShKAI2-3, which were shown to bind KAR_1_. This residue is anticipated to keep the V-shaped helices in a rigid form through hydrogen bonding with L178 of α3, thereby restricting the size of the entrance and the pocket. However, in SL-binding ShKAI2-4, -5, and -7, the residue at position 150 is a phenylalanine, thus disrupting the hydrogen bond and allowing α1 to orient outwards and enlarge the entrance and pocket size. The authors speculated that this is one change that allows the protein to bind SL substrates (Xu *et al.*, 2018). Most notably, mutations introducing bulkier hydrophobic residues such as L124F, T190F, and C194F could confer KAR_1_ affinity upon ShKAI2-7 while abolishing affinity for SLs. These observations are consistent with an earlier report suggesting that F134 and F194 are important in allowing Arabidopsis KAI2 to bind KAR_1_ through aromatic interactions (Guo *et al.*, 2013).

One difficulty with studying the mode of action of karrikins is that they appear to be largely inactive outside of plant cells. For example, karrikins can induce the degradation of KAI2 when intact seedlings are treated with exogenous KAR_1_ or KAR_2_, but in a cell-free extract KAI2 does not show any decreased stability in the presence of karrikins (Waters *et al.*, 2015*a*). KAR_1_ does not enhance the interaction between MAX2 and KAI2 homologues from *Striga hermonthica* in yeast or in pulldown experiments (Xu *et al.*, 2018). GR24^*ent*-5DS^, a SL analogue that is bioactive through KAI2, will trigger thermal destabilization of purified KAI2, but karrikins are inert in this assay (Waters *et al.*, 2015*b*). There is plenty of evidence based on isothermal calorimetry and microdialysis that KAI2 binds KAR_1_, and it is possible to modulate the binding affinity with targeted mutations (Guo *et al.*, 2013; Xu *et al.*, 2016; Lee *et al.*, 2018; Xu *et al.*, 2018; Bürger *et al.*, 2019). However, binding of a hydrophobic compound to a hydrophobic pocket does not equate to receptor activation, and there is disagreement about how karrikins fit into the active site of the receptor protein, with non-congruent orientations in two different crystal structures (Guo *et al.*, 2013; Xu *et al.*, 2016). Admittedly, these two structures are of KAI2 homologues from different species (Arabidopsis and *S. hermonthica*), but collectively these observations do not favour a simple ligand–receptor interaction model for karrikins. It is possible that karrikins are metabolized and activated *in vivo*, or that a complete protein signalling complex is required to stabilize the binding of karrikin to its receptor. Accordingly, although we are now starting to understand how ligand specificity is achieved, we still lack key information about how KAI2 and karrikins work. No doubt this problem in part reflects the fact that karrikins are suboptimal substrates for KAI2.

## After the receptor: structural insights from strigolactones

Currently there is a paucity of structural information about how karrikin–KAI2 signalling operates immediately after ligand perception, but genetic evidence supports a model very similar to that for the SL receptor D14 (see Box 1). Structural evidence for the D14–D3 relationship is strong but there are two alternative models for how these proteins interact. In the first, the lid domain of D14 partially collapses following ligand hydrolysis, and this conformational change is stabilized by D3, thereby trapping a ligand hydrolysis product inside the D14 pocket (Yao *et al.*, 2016). This model is consistent with the idea that D14 is a single turnover enzyme (de Saint Germain *et al.*, 2016), but inconsistent with the fact that SL hydrolysis is dispensable for signalling (see Box 3). In this model, the conformational change may provide a new surface interface for recruitment of SMXL proteins into the ubiquitin ligase complex. In a more recent work, Shabek *et al.* (2018) described how the C-terminal α helix domain of D3 can adopt either an open or a closed state, and that the open state can directly bind D14 in the presence of the SL analogue GR24. Notably, this interaction can inhibit SL hydrolysis by D14, and the presence of SMXL proteins (such as D53 from rice) can restore ligand hydrolysis by D14. The authors proposed that the open form of D3 might provide an interface for D53 recruitment, which would enable their ubiquitination and degradation (Shabek *et al.*, 2018). Although quite distinct, these two models may not be mutually exclusive, as they may represent different stages in a complex signalling process. For example, SMXL repressor proteins may be targeted for ubiquitin-mediated proteasomal degradation first, followed by the D14 receptor; each of these degradation steps might require a different spatial relationship between MAX2, D14, SMXLs and the remainder of the ubiquitin ligase complex.

It will be very satisfying to see whether karrikin–KAI2 signalling operates in the same way as SL–D14 signalling. However, some experimental evidence already indicates that there will likely be some important differences; for example, SL-induced degradation of D14 is dependent on MAX2, but karrikin-induced degradation of KAI2 is MAX2-independent (Chevalier *et al.*, 2014; Waters *et al.*, 2015*a*). Such work may be facilitated by finding compounds that are more suitable KAI2 ligands.

## Conclusions

Karrikin–KAI2 signalling is emerging as a ubiquitous, multi-faceted feature of plant development. The rapid progress in this field over the past 10 years or so is a classic example of what can emerge from curiosity-driven, hypothesis-led science. Looking to the future, there is the clear expectation of discovering a new plant hormone, which, as with the identification of strigolactones as plant hormones, will be very challenging and require innovative, multidisciplinary approaches. A further challenge will be to document the extent of KAI2-related phenomena and how they differ across species. How does KAI2 signalling facilitate arbuscular mycorrhizal symbiosis? How do different SMXL proteins confer different downstream responses? Are karrikins metabolized into an active compound, and through what mechanism? And how can we best translate our discoveries into meaningful benefits for crop breeders and farmers worldwide? With new tools—and increasingly collaborative researchers—answering these questions will soon become feasible.

**Box 1. Key developments in understanding karrikin function in plants**

**• KAI2 promotes plant tolerance to drought**

Li *et al.* (2017) reported that KAI2 is important for plant responses to drought. They reasoned that KAI2-mediated drought adaptation has three components: (i) KAI2 promotes ABA catabolism; *kai2* mutants have higher ABA content and reduced ABA response, leading to enlarged stomatal apertures. (ii) KAI2 promotes anthocyanin biosynthesis; *kai2* mutants fail to accumulate anthocyanin, which offers protection from reactive oxygen species associated with many types of abiotic stress. (iii) KAI2 promotes the formation of the cuticle; *kai2* mutants have a thinner cuticle, while KAI2 overexpressors have a thicker cuticle. The authors did not directly test whether exogenously applied karrikins would induce drought tolerance (Li *et al.*, 2017).

**• The KAI2 signalling pathway regulates root development**

Two publications (Swarbreck *et al.*, 2019; Villaécija-Aguilar *et al.*, 2019) reported the effects of KAI2–SMAX signalling on root development in Arabidopsis. KAI2-mediated signalling controls root skewing (angle relative to vertical), root hair density and root hair length, and in conjunction with D14-mediated signalling, also regulates lateral root density. There is some disagreement about whether all of the KAI2-dependent effects are mediated by SMAX1 and SMXL2 as the canonical signalling model would predict (see Box 2), but Villaécija-Aguilar *et al.* (2019) report that changes in growth conditions between laboratories apparently influence root skewing, and the role of SMXL6, 7, and 8 in regulating this phenotype.

**• A modified karrikin response under abiotic stress**

Wang *et al.* (2018) reported an interesting phenomenon in which abiotic stress—for example salinity or osmotic stress—can change karrikins from being a positive regulator of germination to an inhibitor. Under such conditions, karrikin can also promote transcription of genes encoding stress response transcription factors like *WRKY33*, *ERF5*, and *DREB2A* in a KAI2-dependent manner. The authors proposed that KAI2 can serve as a stress sensor so that the presence of karrikins can prevent seeds from germinating under unfavourable conditions. However, the mechanism behind this reversal is not understood (Wang *et al.*, 2018).

**• Rigidity between helices α2 and α3 in KAI2 contributes to ligand affinity**

The moss *Physcomitrella patens* does not show growth responses to karrikins (Hoffmann *et al.*, 2014) but the genome nonetheless encodes 11 KAI2 homologues, raising the question of when karrikin perception evolved. In a detailed structural study of these moss homologues, Bürger *et al.* (2019) found a subset of PpKAI2 homologues that could bind KAR_1_, while others could bind to synthetic SLs with opposite stereochemistry to natural SLs. The authors proposed that a rigid loop linking helices α2/α3 is important for SL affinity, by constricting the size of the ‘tunnel’ that allows access to the catalytic site. PpKAI2 proteins could not complement the Arabidopsis *kai2* mutant phenotype, making it difficult to conclude whether KAR_1_-binding PpKAI2 homologues can really transduce a karrikin signal (Bürger *et al.*, 2019).

**Box 2. Karrikin and strigolactone signalling components**

Genetic studies in Arabidopsis, rice, pea, petunia, and more have three functionally conserved groups of proteins essential for karrikin and strigolactone signalling. Karrikins are perceived through the α/β hydrolase KARRIKIN INSENSITIVE2 (KAI2; also known as HTL), while strigolactones are perceived through a paralogous protein, DWARF14 (D14; also known as DAD2 in petunia and RMS3 in pea), that evolved in seed plants. Both are functional hydrolases with a hydrophobic ligand-binding pocket. These proteins appear to be metastable and prone to conformational changes associated with ligand binding, though the exact significance of hydrolysis is still debatable (see Box 3). The second key protein, which is common to both karrikin and SL signalling pathways, is MORE AXILLARY BRANCHES2 (MAX2; D3 in rice). MAX2 is an F-box protein that forms part of the Skp–Cullin–F-box class of E3 ubiquitin ligases; its role is to ubiquitinate specific target proteins for proteasomal degradation. These target proteins form the third class of signalling component in the SUPPRESSOR OF MAX2-LIKE (SMXL) family, which are related to ATP-dependent heat-shock proteins and likely operate as transcriptional corepressors. In most land plants, especially in angiosperms, there has been extensive gene duplication and functional diversification within the SMXL family. In simple terms, a subset of SMXL proteins (the most evolutionarily ancient ones represented by SMAX1 and SMXL2 in Arabidopsis, and OsSMAX1 in rice) bring about KAI2-dependent responses. Another group (represented by D53 in rice and SMXL6, SMXL7, and SMXL8 in Arabidopsis) mediate SL-dependent responses. Thus karrikin and strigolactone signalling use very closely related componentry with specificity at both input and output levels. Figure modified from Waters (2017), *Functional Plant Biology* 44(4), 373–385.

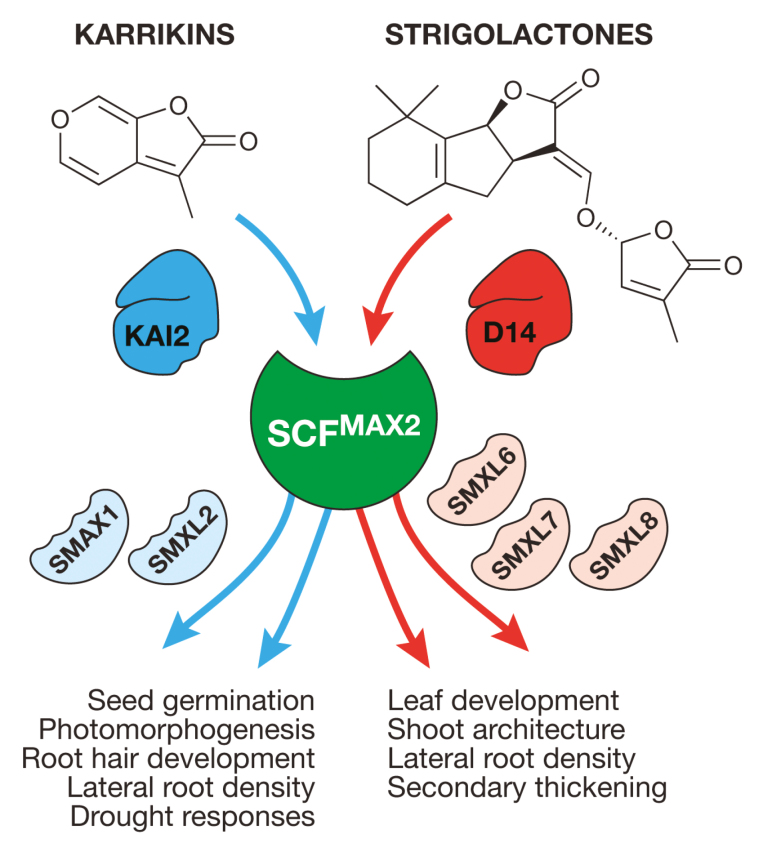

Box 3. **The functional significance of ligand hydrolysis**

As α/β hydrolases, KAI2 and D14 both possess hydrolytic activity towards a variety of butenolide substrates thanks to a conserved catalytic triad of Ser, His, and Asp residues. However, the necessity of this activity for signalling is unclear. The likely mode of action for ligand hydrolysis is nucleophilic attack by the catalytic Ser upon the butenolide carbonyl group of the ligand, followed by a transient ligand–receptor intermediate where a portion of the butenolide moiety is covalently attached via the His residue (de Saint Germain *et al.*, 2016; Yao *et al.*, 2016). This covalent intermediate may be associated with a conformational change in the receptor protein (de Saint Germain *et al.*, 2016; Hamiaux *et al.*, 2012), which in turn is stabilized by interaction partners such as MAX2 (Yao *et al.*, 2016). In the case of D14, mutation of the catalytic Ser or His residues abolishes hydrolytic activity and biological function, suggesting that hydrolysis is essential for signalling (Hamiaux *et al.*, 2012; Nakamura *et al.*, 2013). However, a recent publication brings this orthodoxy into doubt. Seto *et al.* (2019) reported that mutation of the Asp residue does not affect the capacity of D14 to transduce a SL signal, even though hydrolytic activity itself is abolished. One explanation for this unexpected result could be that Ser and His are required for ligand binding, whereas Asp is not: in the case of rice D14, the Ser and His residues are located at the surface of the hydrophobic pocket close to the presumed ligand binding site, but the Asp residue is embedded on a loop relatively distant from the ligand (see figure). Seto *et al.* (2019) propose that the Asp-containing loop plays a critical role in protein conformational change, while ligand hydrolysis is a slow process that deactivates the SL ligand after the receptor has been activated: that is, hydrolysis is not a requirement for signalling (Seto *et al.*, 2019).

However, there are two observations that are inconsistent with this latter interpretation. This first is the invariant evolutionary conservation of the catalytic residues. D14 protein is degraded as a result of signalling, in a MAX2-dependent manner (Chevalier *et al.*, 2014), so it is not immediately obvious why the ligand should be deactivated by hydrolysis when the receptor itself will not be recycled. If hydrolysis were not crucial for signalling, then we should find natural D14 variants lacking the Asp residue. Second, butenolide ligands that are non-bioactive as SLs are hydrolysed very rapidly compared to bioactive ligands (de Saint Germain *et al.*, 2016). This suggests that bioactivity might depend upon slow hydrolysis, perhaps because of the need for sufficient time for a covalent intermediate to bring about protein conformational change thought necessary for signalling.

For KAI2, the catalytic Ser residue is essential for function *in vivo* (Waters *et al.*, 2015b), but the functional requirement for the other two residues has not yet been described. The real enigma with KAI2 is how karrikins themselves work: karrikins are not thought to be susceptible to hydrolysis in the same way as SL molecules, because karrikins lack a suitable leaving group that would allow the butenolide moiety to remain open after nucleophilic attack (Scaffidi *et al.*, 2012). That said, if ligand hydrolysis is not necessary for signalling via KAI2 as appears to be the case for D14, then the lack of karrikin hydrolysis is no longer problematic.

**Figure caption:**

Rice D14 in complex with synthetic SL analogue GR24^5DS^. The ligand sits within the main pocket (surface shown in grey), at the bottom of which lies the Ser–His–Asp catalytic triad. In this structure, the ligand just protrudes from the protein surface. The four α helices of the lid domain are shown in cyan, and the core domain in green. Images created in PyMol v2.2.0 and based on PDB code 5DJ5 (Zhao *et al.*, 2015).